# Metal chelation as an antibacterial strategy for *Pseudomonas aeruginosa* and *Acinetobacter baumannii*

**DOI:** 10.1039/d4cb00175c

**Published:** 2024-09-24

**Authors:** Martina M. Golden, Amelia C. Heppe, Cassandra L. Zaremba, William M. Wuest

**Affiliations:** a Department of Chemistry, Emory University Atlanta GA 30322 USA wwuest@emory.edu; b Emory Antibiotic Resistance Center, Emory School of Medicine, Emory University Atlanta GA 30322 USA; c Department of Chemistry and Biochemistry, Denison University Granville OH 43023 USA zarembac@denison.edu

## Abstract

It is estimated that by 2050, bacterial infections will cause 1.8 million more deaths than cancer annually, and the current lack of antibiotic drug discovery is only exacerbating the crisis. Two pathogens in particular, Gram-negative bacteria *A. baumannii* and *P. aeruginosa*, are of grave concern because of their heightened multi-drug resistance due to a dense, impermeable outer membrane. However, targeting specific cellular processes may prove successful in overcoming bacterial resistance. This review will concentrate on a novel approach to combatting pathogenicity by disarming bacteria through the disruption of metal homeostasis to reduce virulence and enhance antibiotic uptake. The varying levels of success in bringing metallophores to clinical trials, with currently only one FDA-approved siderophore antibiotic to date, will also be detailed.

## Abstract 2

*A. baumannii* and *P. aeruginosa* pose serious concerns due to heighted multi-drug resistance. A novel method to reduce bacterial virulence and enhance the effectiveness of antibiotics may be achieved by disrupting native metal homeostasis.

## Graphical abstract

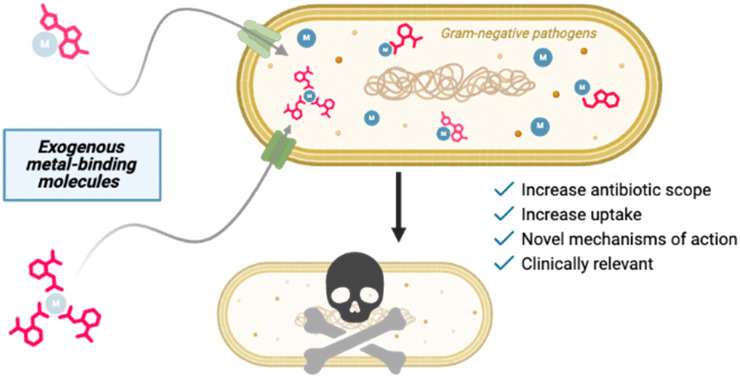

## Introduction

In 2019, one out of every eight deaths were accredited to a bacterial infection, which made these infections the second leading cause of death in the world.^1^ Many of these bacteria are resistant to our current antibiotics, and this problem has only been intensified since the COVID-19 pandemic. Specifically, it was noted that 80% of patients hospitalized with COVID-19 were prescribed antibiotics, and antibiotic use in nursing homes increased 5% from pre-pandemic levels.^2^ This overuse is an attributing factor to increased levels of resistance worldwide. As shown in the UK, the number of bacterial infections and related deaths is growing every year, with a notable 4% increase in antibiotic-resistant infections from 2021 to 2022.^3^ The antibiotic resistance crisis is due to many factors including unnecessary overprescription, inappropriate livestock usage, and a lack of drug discovery within the field of antimicrobial resistance (AMR).^4,5^

To focus our efforts towards developing the most relevant antimicrobials, the World Health Organization (WHO) and the Center for Disease Control (CDC) publish lists of the most concerning microorganisms for human health. The CDC coined the term “ESKAPE” pathogens for the bacteria that have the highest level of potential to develop further antibiotic resistance.^6^ Those six bacteria are: *Enterococcus faecium*, *Staphylococcus aureus*, *Klebsiella pneumoniae*, *Acinetobacter baumannii*, *Pseudomonas aeruginosa*, and *Enterobacter* species, and they can be found in up to 97% of clinical isolates of bacteria.^6,7^ Of the six ESKAPE pathogens, four are Gram-negative bacteria (*K. pneumoniae*, *A. baumannii*, *P. aeruginosa*, and *Enterobacter* species).

**Fig. 1:**
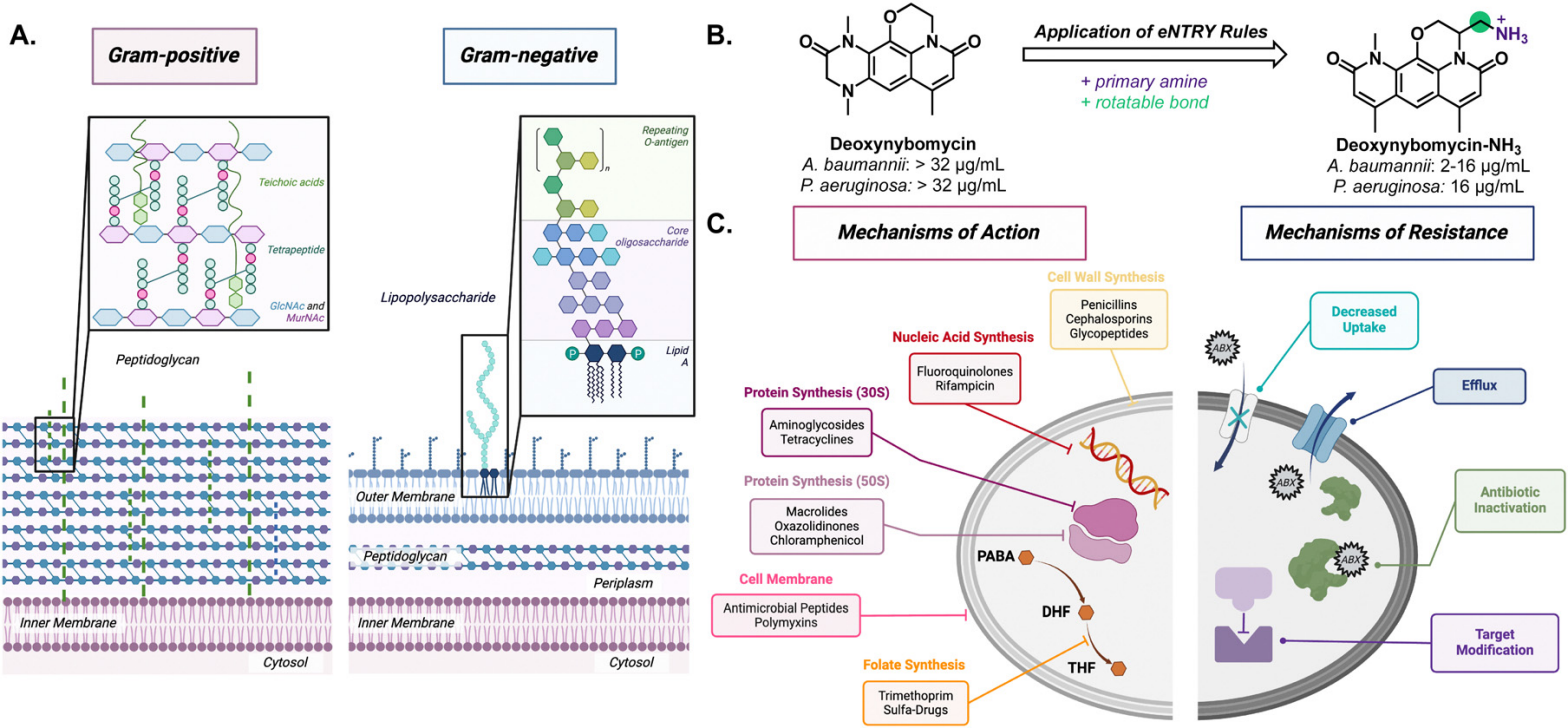
(A) Comparison between single membrane Gram-positive and the inner and outer membrane of Gram-negative bacteria. (B) Example of using the eNTRY rules to increase antibiotic activity in Gram-negative bacteria. (C) Canonical antibiotic mechanisms targeting essential cellular processes and the bacteria's native and adapted mechanisms of resistance.

Gram-negative bacteria pose a particular challenge in fighting AMR due to its secondary membrane and lipopolysaccharide (LPS) exterior (Fig. 1(A)). The LPS creates an exceptionally impermeable barrier with densely packed, negatively charged subunits stabilized by divalent cations such as Mg^2+^ and Ca^2+^.^8^ This makes diffusion of small molecules, especially nonpolar molecules, across the outer membrane rather difficult. Most molecules gain entry through porins *via* passive diffusion. However, these channels are often lined with negatively charged amino acids that impede nonpolar small molecule diffusion.^9^ Even if small molecule antibiotics make it through the outer membrane, active transport efflux pumps can still remove unwanted material out of the bacteria.^10^

Through structure–activity relationship studies, it has been postulated that compounds with certain properties will penetrate Gram-negative bacteria if they follow “eNTRy rules.”^11^ The eNTRY rules describe three conditions that have been shown to increase antibiotic permeability in Gram-negative bacteria: ionizable nitrogen, lack of three-dimensionality, and high rigidity, and these rules have shown great success in increasing antibacterial activity against *E. coli*. For example, they are able to increase the cellular accumulation of the Gram-positive antibiotic, deoxynybomycin, through the addition of a primary amine by fourfold; thus, increasing the scope for the antibiotic (Fig. 1(B)). This alteration brought the minimum inhibitory concentration (MIC) from >32 μg mL^−1^ to 0.5–16 μg mL^−1^ in *E. coli*, *A. baumannii*, and *K. pneumoniae.*^12^ More recently, the eNTRY rules were amended to better represent *P. aeruginosa* showing that the primary amine was the only indicator and that there was not a strong trend with other structural features.^13^ Although improvements, such as the one to deoxnybomycin, have been made to increase the scope of antibiotics, there still exists a significant lack of innovation in antibiotic discovery.

The root of the problem is that most antibiotics have the same mechanism of action; wherein they target the synthesis of cellular components (proteins, nucleic acids, or cell wall) (Fig. 1(C)). In fact, the FDA reported that just 50% of experimental therapies, designated as Qualified Infectious Disease Products (QIDPs) acted in a novel mechanism of action.^14^ As a result, many strains have developed resistance against these common mechanisms and show cross-resistance across different families of antibiotics.^15^ Through fast replication rates and horizontal gene transfer, bacteria can easily undergo adaptive evolution to become resistant to even the strongest of antibiotics.^4^ The combination of cell impermeability and heightened multidrug resistance has made these ESKAPE pathogens a growing concern to healthy and immunocompromised patients around the world.

Unfortunately, the path toward successful antibiotic drug discovery is challenging and lengthy. Bringing a commercial-ready antibiotic to market can take 15–20 years and hundreds of millions of dollars.^16–18^ Additionally, many large pharmaceutical companies have abandoned their AMR programs in favor of more profitable drug therapies.^19^

To this end, strategies are being employed to circumvent the long development time for bringing new drugs to market and the quick time to resistance emergence. For example, combining specific antibiotics in treatment may lead to a synergistic effect.^20^ Another strategy is drug repurposing, where previously FDA-approved drugs are analyzed for antimicrobial properties.^21–23^ Described herein is a different strategy in which metal homeostasis is targeted rather than bacteria's vital cellular processes. Metals play an important role in the regulation of other virulence factors (*e.g.* biofilms and quorum sensing), bacterial physiology and pathogenesis. In this case, the bacteria's mechanism of infection and transmission can be hindered and their ability to cause and exacerbate disease may be inhibited.^24^ Targeting virulence factors represents a novel mechanism of action in antibiotics, broadening the diversity of the current arsenal.

This review will focus on two of the most concerning Gram-negative ESKAPE pathogens: *P. aeruginosa* and *A. baumannii*.^25^ These two represent a large portion of nosocomial infections due to their multitude of intrinsic resistance mechanisms and virulence factors. To combat this issue, innovative antibacterials for these pathogens are of utmost importance. One promising method would be exploiting bacterial metal homeostasis given its important role in bacterial survival and virulence. We will explore how these pathogens sequester metals as well as the ways in which nature and scientists have exploited those mechanisms for new antibacterials.

### Role of metals in bacterial pathogenesis

**Fig. 2:**
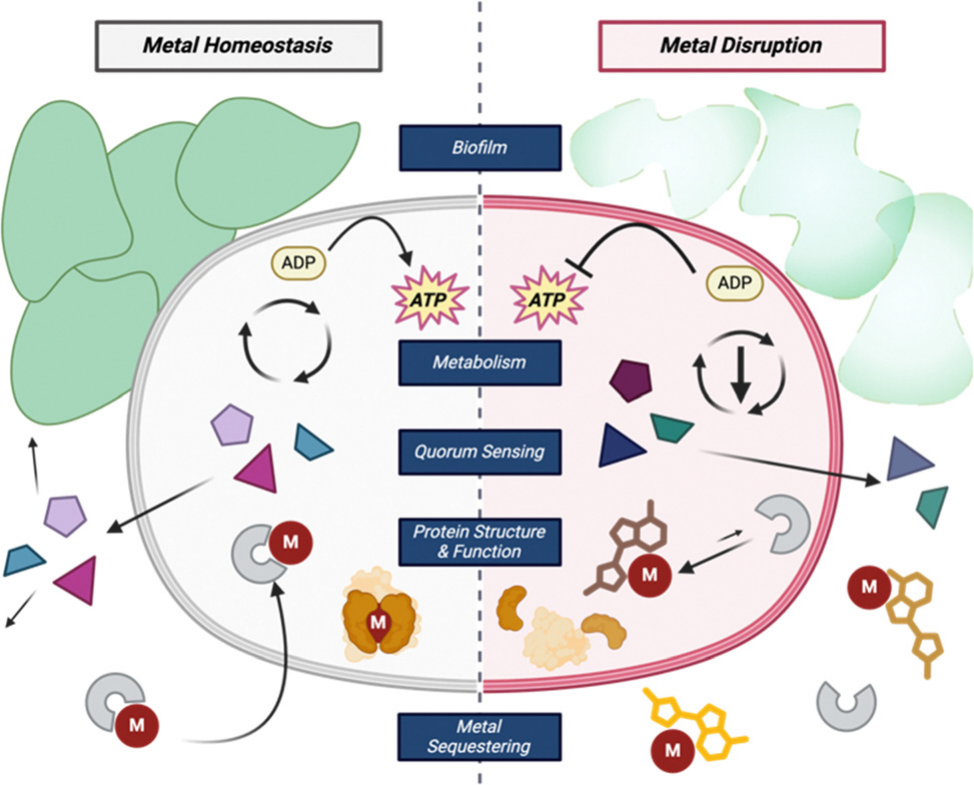
Common roles of metals within bacterial cells and how disrupting those processes can affect the cell.

Metals play an essential role in maintaining homeostasis in prokaryotes and eukaryotes alike. Metalloenzymes make up one-third of all enzymes and can catalyze a wide variety of biological reactions.^26^ The purpose of a metal within an enzyme can range from providing a central ligand binding site, acting as a Lewis acid, or operating as a reducing agent in various cellular oxidation–reduction reactions. Metal deficiencies can lead to decreased enzyme activity, energy production, and weaker cells (Fig. 2).^27^ To maintain metal homeostasis, metalloregulatory proteins regulate gene expression of metal-dependent processes, efflux pumps, metallochaperone proteins, and the biosynthesis of siderophores.^28,29^

Metal acquisition is not only a key part of metabolism but is also a virulence factor in pathogenic bacteria. There exists a constant battle between the animal host and the bacteria for iron; both animals and bacteria alike have certain molecules to aid in “stealing” the limited iron resource away from the other. In an iron-deficient infection ([Fe^3+^] = 10^−24^ M),^30^ bacteria will compete with the host to sequester iron to increase its own iron concentration to 10^−6^ M.^31^ Specifically, bacteria use molecules called siderophores, from the Latin root words *sideros* and *phoros* meaning iron and carrier, respectively, and have a high affinity for chelating Fe^3+^.^32^ These are synthesized intracellularly and then excreted into the extracellular matrix to capture free iron from the environment or sequester it away from other metal complexes. In addition to their native siderophore complexes, bacteria can uptake exogenous metal complexes which creates interspecies competition for this valuable resource.

The exact relationship between metallophores and virulence factors like biofilm formation within ESKAPE pathogens remains unclear, but metal disruption certainly impacts the bacteria's pathogenicity.^33–37^ Antibiotic discovery that focuses on inhibiting the expression of virulence is particularly important for disarming pathogenesis mechanisms and limiting the severity of infection. Below, we will highlight some of the natural products that utilize metal binding motifs to exert antibacterial activity.

## Metal binding small molecules

Native metallophores are molecules with a high affinity for their metal of interest and have a low molecular weight.^38^ They have key structural motifs that participate in metal binding which usually involve heteroatoms, specifically oxygen, nitrogen, and sulfur. Some of the canonical structural motifs in siderophores include phenolates, catecholates, hydroxamates, hydroxylamines, carboxylates, and thiazoline/oxazoline rings. In addition to the functional groups that can bind iron, copper ligands frequently have isonitrile functional groups. Most commonly, these functional groups form an octahedral geometry around the metal.^39^ Fortunately for chemists, the same functional groups are used for metal binding across the tree of life which facilitates fast determinations of whether a structure is likely to bind metals and hypothesize potential structure–activity relationships. Siderophores and their mechanisms of biosynthesis, metal binding, and uptake have been well studied.^32,40,41^ This has made them the primary ligand for antibacterial metallophores; however, a wide range of metals are required for biology and how bacteria acquire and utilize these metals is less understood.

Native siderophore production in many bacteria, including *P. aeruginosa* and *A. baumannii*, is regulated by a ferric uptake regulator (Fur) protein.^42,43^ Fur binds to iron-regulated promoters thus acting as a transcriptional repressor of the genes for biosynthesis of siderophores. When iron levels are sufficient, Fur binds Fe^2+^, and the conformation of the metalloprotein allows it to bind to target DNA sequences. However, when iron levels decrease, the equilibrium shifts, and Fur will release its Fe^2+^ allowing expression of the previously repressed genes. The details of this process have been reviewed previously by Escolar *et al.*^44^ Upon biosynthesis of siderophores, they will be transported out of the cell to sequester iron, usually in the Fe^3+^ oxidation state. The metal complex will be actively transported through the outer membrane and into the periplasm *via* a TonB-dependent transporter (TBDT) where it will be reduced to Fe^2+^ for incorporation into cellular processes.^41^

### *Pseudomonas aeruginosa* siderophores

**Fig. 3:**
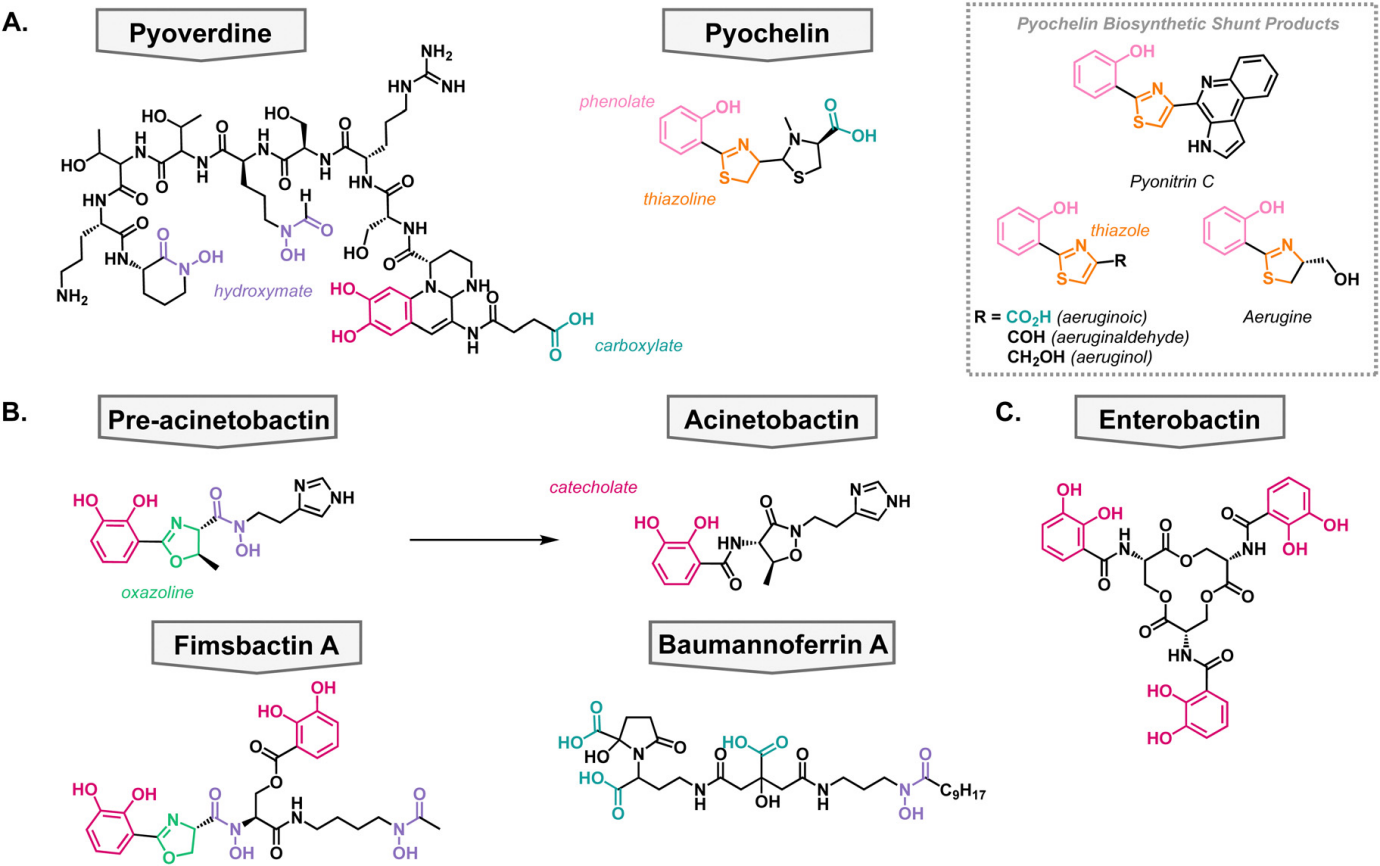
Native metallophores in *P. aeruginosa* and *A. baumannii*. Common metal binding functional groups are color-coordinated throughout the review. Magenta: catecholate, pink: phenolate, purple: hydroxymate, teal: carboxylate, orange: thiazoline and thiazole, green: oxazoline. (A) *P. aeruginosa* siderophores. (B) *A. baumannii* siderophores. (C) Xenosiderophore taken up by both bacteria.

*P. aeruginosa* primarily synthesizes two siderophores; pyoverdine which has a high affinity for iron (pFe = 27) and pyochelin which has a lower affinity for iron (pFe = 16) but requires fewer resources to synthesize (Fig. 3(A)).^45–47^ It has been shown that *P. aeruginosa* first synthesizes pyochelin and will only switch to synthesizing pyoverdine when iron levels become very low.^48^ This can be advantageous when competing for nutrients in a polymicrobial environment, which is frequently the case when establishing an infection. The two native siderophores have differing TBDTs. Pyochelin is recognized and transported by FptA,^49,50^ and pyoverdine is recognized and transported by two proteins: FpvA and FpvB.^51,52^ All of these TBDTs utilize the proton motive force for active transport into the periplasm.

In the biosynthesis of pyochelin, a variety of shunt products are made which exhibit different oxidation states of pyochelin.^53–55^ These products have been shown to also be able to bind iron, albeit with less affinity than pyochelin (Fig. 3(A)).^56^ Interestingly, the shunt products bind iron in varying metal-to-ligand ratios in comparison to pyochelin which binds Fe^3+^ in a one-to-one ratio. To further probe the structure activity relationship of pyochelin and synthetic analogs, a panel of analogs were synthesized wherein the thioazoline was converted to an oxazoline ring.^57^ This is a common motif in siderophores from other species and would assess the promiscuity of *P. aeruginosa's* iron uptake mechanisms.^40^

All pyochelin analogs were pre-chelated to iron and the growth of *P. aeruginosa* in iron-deficient media. The compounds promoted the growth of *Pseudomonas* suggesting that they can be taken up by the cell similarly to traditional siderophores. Moreover, these substitutions did not inhibit the growth of any of the *Pseudomonas* strains tested nor any other pathogens, suggesting that it does not act as an antibacterial metallophore. This could give *Pseudomonas* an upper hand in nature since it can acquire iron from the side products of siderophore biosynthesis in addition to the native siderophores. In summary, this shows that there is a structure activity relationship to the molecules that *Pseudomonas* produces, but the uptake machinery is promiscuous and will accept a variety of metal complexes.

### *Acinetobacter baumannii* siderophores

Contrary to *P. aeruginosa*, *A. baumannii* utilizes both species-specific siderophores in addition to the uptake of broad-spectrum siderophores, usually produced by another organism. On the species-specific side, *A. baumannii's* siderophores fall into three classes: acinetobactins, fimbactins, and baumannoferrins. The most well-known of *A. baumannii*'s siderophores is acinetobactin, which comes from the precursor pre-acinetobactin, both of which act as species-specific siderophores. They are synthesized *via* a nonribosomal peptide synthetase, and the structures convert nonenzymatically and are pH dependent.^58,59^ Where acinetobactin is the active siderophore in basic conditions (high pH) and pre-acinetobactin is the active siderophore in acidic conditions (low pH, Fig. 3(B)).^59^ This is an important feature of the siderophore since different infection sites may vary pH, and acinetobactin biosynthesis has been shown to be necessary for virulent mouse infection models.^60^ Once bound to iron, the pre-acinetobactin complex will be transported into the periplasm *via* the TBDT BauA, however, BauA does not recognize acinetobactin.^61,62^ Moreover, it is proposed that pre-acinetobactin is the primary iron deliverer for *A. baumannii* while acinetobactin is an alternative carrier that funnels sequestered iron to pre-acinetobactin in a siderophore shuttle mechanism.^63–65^

Knowing that *A. baumannii* strains grow in the absence of acinetobactin biosynthesis, it was hypothesized that it produced other siderophores, besides the acinetobactins. In 2013, the fimsbactin family of siderophores was discovered by growing *Acinetobacter* species in iron-limited media (Fig. 3(B)).^66^ Fimsbactin A accounted for over 85% of the total mass isolated and the rest of the compounds (fimsbactin B–F) are biosynthetic shunt products or intermediates towards the synthesis of fimsbactin A. Subsequently, it was shown that fimsbactin-iron complexes can promote the growth of *A. baumannii* in iron-limited media, but when in combination with acinetobactins, fimsbactin inhibits the growth of *A. baumannii* suggesting that the siderophores compete for a similar pathway.^67^ It is worth noting that only about 10% of *A. baumannii* strains have the biosynthetic genes for fimsbactin *versus* the ubiquitous representation for the acinetobactins. Shortly behind the discovery of fimsbactin, the baumannoferrin siderophores were discovered in *A. baumannii* AYE, a multidrug resistant clinical isolate incapable of synthesizing acinetobactins (Fig. 3(B)).^68,69^ This was an important finding to understand that other siderophores can aid the *A. baumannii* virulence and pathogenesis.

### Small molecule xenosiderophores

In addition to their species-specific siderophores, *P. aeruginosa* and *A. baumannii* will uptake exogenous metal complexes, when necessary; these are called xenosiderophores. This can be beneficial when in a polymicrobial community to avoid utilizing one's own resources to acquire vital nutrients. These pathogens have adapted to stealing iron complexes from the environment as highlighted by *P. aeruginosa's* 34 TBDTs and *A. baumannii's* 21 TBDTs, most of which are hypothesized to be involved in iron acquisition.^70,71^ For example, enterobactin is a siderophore produced by *E. coli* but can be taken up by both *P. aeruginosa* and *A. baumannii* (Fig. 3(C)).^72^ Additionally, enterobactin has been the inspiration for siderophore analogs that have been developed into siderophore–antibiotic conjugates which will be discussed later in the review.

## Antibacterial siderophores

Given that iron binding small molecules are vital for microbe well-being, siderophores are involved in microbe stress response and protection from competitors. In recent years, the molecules that elicit these effects have been elucidated and characterized. Efforts are underway to isolate natural products that have antibacterial activity. This section covers promising siderophores with activity against *P. aeruginosa* and *A. baumannii*, as well as synthetic analogs. The antibacterial siderophores detailed below have various proposed mechanisms of inhibition, ranging from intercellular iron scavenging to xenosiderophore imitation. However, the specific mechanisms are yet to be wholly defined due to highly nuanced and complex metal-binding pathways. More work is certainly necessary to leverage metal homeostasis for bacterial inhibition.

### Cahuitamycins

**Fig. 4:**
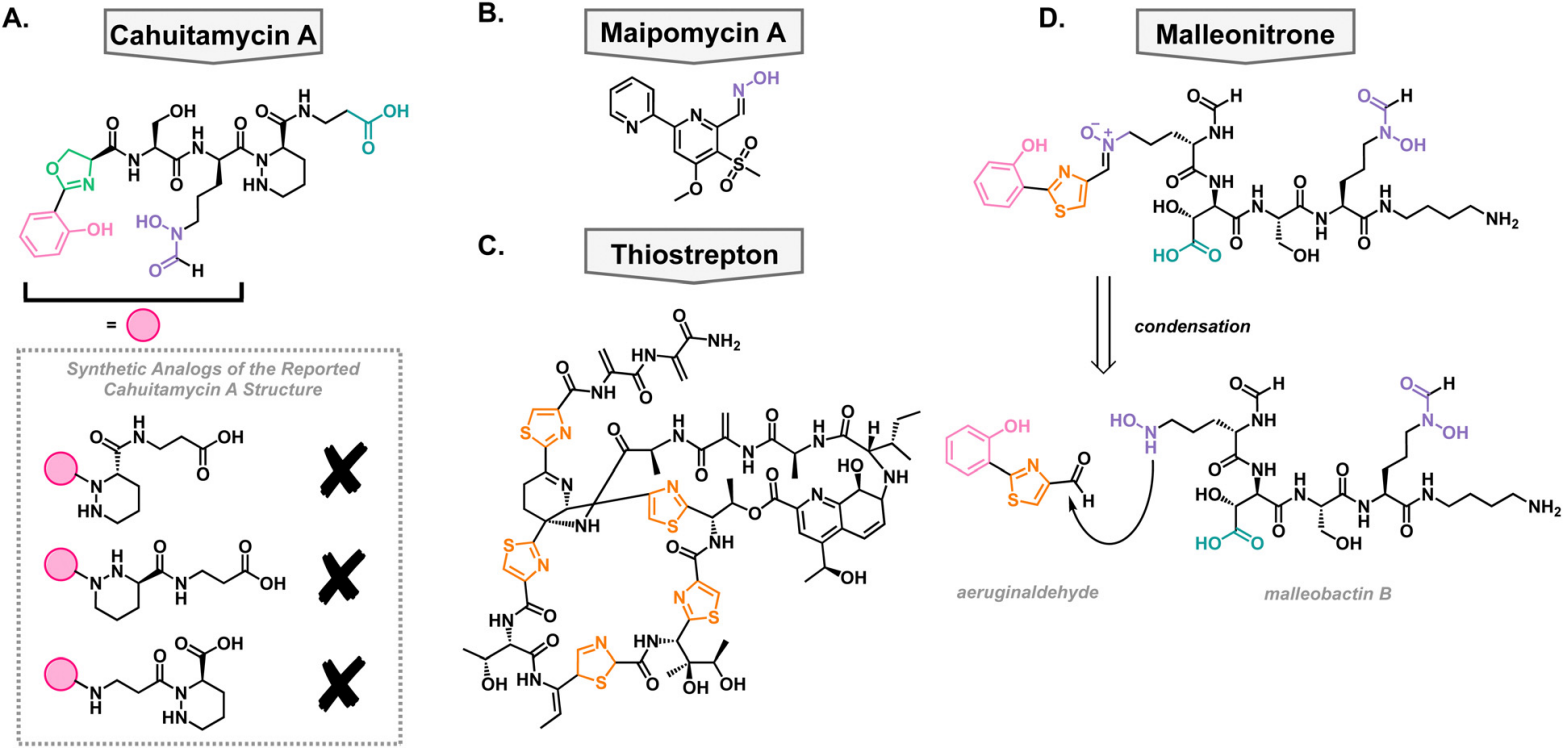
Structures of natural and synthetic antibacterial siderophores. (A) Reported structure of cahuitamycin A and synthetic analogs which are not in agreement with reported data. (B) Maipomycin A. (C) Thiostrepton. (D) Structure of malleonitrone and its proposed retrosynthesis to aeruginaldehyde and malleobactin B.

In a high-throughput assay, a library of natural product extracts was screened for their ability to inhibit *A. baumannii* biofilm formation. This screen successfully obtained an extract from *Streptomyces gandocaensis*, and through extensive ribosomal engineering, they optimized the biosynthesis of the active components. This was isolated and characterized as a nonribosomally synthesized hexapeptide with unique, metal binding residues such as an oxazoline ring, hydroxamates, and a piperazic acid residue.^73^ The active components of this extract were named cahuitamycin A–C (Fig. 4(A)). It was found that cahuitamycin A and C inhibit *A. baumannii* biofilm formation. This biological activity could be attenuated by changing iron concentrations, suggesting a relationship between its mechanism of action and iron binding motifs. Interestingly, it was found that cahuitamycin C can do this without significant planktonic growth inhibition. This is an important characteristic for a potential virulence inhibitor to prevent selection for resistant strains of bacteria.

Due to the unique structure and bioactivity of the cahuitamycin family of natural products, they have been a target molecule for many research groups, including our own. The total synthesis of this family has been stalled due to inconsistencies between the reported structure of cahuitamycin A and the reported chemical and biological characterization data.^74^ With a sample of authentic cahuitamycin A in hand, Shapiro and coworkers performed a variety of spectroscopic experiments finding some key discrepancies near the reported piperazic acid region of the natural product, the synthetic version, and various analogs of the compound (Fig. 4(A)). This was further confirmed when synthetic cahuitamycin A, nor any analogs synthesized by our group, failed to inhibit planktonic growth of *A. baumannii*, as the isolation report stated. To date, the true structure of cahuitamycin A has yet to be discovered, despite studies of the biosynthetic gene cluster. To fully understand its activity against *A. baumannii* and potential as a virulence inhibitor, it will be necessary to determine the true structure of cahuitamycin A.

### Maipomycin A

In an effort to discover a broad-spectrum biofilm inhibitor, Zhang and coworkers analyzed maipomycin A (MaiA) which was isolated from a rare actinomycete strain *Kibdelosporangium phytohabitans* XY-R10 in 2021 (Fig. 4(B)).^75^ The authors found that it displays potent biofilm inhibition of two of the most concerning pathogens, *A. baumannii* and *P. aeruginosa*, without any reported cytotoxic effects towards mammalian cells. MaiA is able to bind Fe^3+^ and Fe^2+^, and its ability to inhibit biofilm is antagonized by the addition of iron showing that it is necessary for its mechanism of action. However, other iron chelators do not show the same anti-biofilm effects indicating that MaiA's mechanism of action is more nuanced than simple iron binding. Furthermore, it was shown that MaiA reduces overall metabolism in *A. baumannii* and *P. aeruginosa* suggesting intracellular iron scavenging. The authors performed thorough structural studies *via* NMR and X-ray crystallography and determined the structure to be a 2,2′-bipyridine system with an aldoxime and novel sulphone moiety. Compounds with related structures have been isolated before and show varying biological activities which exemplifies how slight changes in the structure of these compounds can alter the biological activity.

### Thiostrepton

In 2019, the Burrows lab performed a high-throughput screen of FDA-approved off-patent drugs and found that thiostrepton promoted biofilm production in *P. aeruginosa* (Fig. 4(C)).^76^ Upon further dose–response experimentation, they showed that in addition to promoting biofilm production, it also decreased planktonic cell growth when grown in dilute growth medium (10% lysogeny broth). These were surprising results given that it was well accepted that thiostrepton was exclusively a Gram-positive antibiotic, likely due to poor outer membrane permeability.^77,78^ They concluded that in the restricted media, there must be a reason that *P. aeruginosa* is sensitized to thiostrepton. They were able to rule out amino acid sequestration and turned to siderophore uptake. This idea was supported by a synergistic relationship between thiostrepton and the FDA-approved iron-chelated deferiprone and deferasirox suggesting that its mechanism of action is iron dependent. To determine how it was entering the cell, a transposon library of *P. aeruginosa* siderophore receptors was screened against thiostrepton as well as mutants without FpvA and FpvB, the pyoverdine TBDTs, that showed decreased susceptibly to thiostrepton. This suggested that thiostrepton was utilizing siderophore machinery to gain entry. However, this was unexpected as they showed that thiostrepton does not bind iron, based on the qualitative color change of chrome azurol S agar. Therefore, it is likely not supplying the bacteria any iron but rather imitating the structure of xenosiderophores for uptake into the cell wherein it can exert its killing effect.

In addition to its antibacterial activity against lab strains of *P. aeruginosa*, it was found to be active against clinical isolates of the pathogen which was enhanced by the combination with deferasirox. Given that *A. baumannii* has homologues of FpvA and FpvB, it was also tested against thiostrepton.^76^ Despite being less effective, it was able to inhibit the growth of both lab strains and clinical isolates. This suggests that targeting these outer membrane transporters could be an effective way of making narrow-spectrum antibiotics and that the growth conditions can influence whether a compound will be effective against a pathogen.

### Malleonitrone

In addition to independent synthesis of metallophores, it has been shown that metabolites from different species can react to generate novel structures. In a study employing *P. aeruginosa* and *Burkholderia thailandensis*, it was found that aeruginaldehyde, a pyochelin by-product, reacts with malleobactin B, a *B. thailandensis* siderophore, through a condensation reaction with subsequent oxidation to the thiazoline ring (Fig. 4(D)). The product of this reaction was named malleonitrione which exerts antibacterial activity towards *P. aeruginosa* with an MIC of 25 μg mL^−1^.^79^ Currently, malleonitrione's mechanism of action is unknown but it would be reasonable to hypothesize that it is being taken up by *P. aeruginosa* with the same machinery as pyochelin, due to their structural similarities. The mechanism of antibacterial activity necessitates further investigation. With the prevalence of polymicrobial infections, this work highlights the importance of studying the interactions of those communities and interspecies chemical warfare as it can lead to the discovery of new antimicrobials.

## Antibacterial chalkophores

For decades, siderophores have been the center of attention in bacterial metallophores. However, there are other biologically relevant metals that bacteria require which can be exploited for antimicrobial applications. Copper has received heightened attention lately due to the increased understanding of natural ligands and its role in bacterial metabolism. Compounds that bind copper are called chalkophores, originating from Greek “chalkos” meaning copper.^80^ One of the most well-studied chalkophores is the methanobactin family which comes from methanotrophic bacteria.^81^ However, their antibacterial activity is limited to Gram-positive bacteria which is beyond the scope of this review.^82^ The chalkophore yersiniabactin has been studied further in recent years, and new biosynthetic origins and molecular interactions have been elucidated but, as of now, no antibacterial activity towards *A. baumannii* or *P. aeruginosa* has been reported. Below are molecules that display a range of antibacterial activities through novel and unknown mechanisms of action.

### Xanthocillin X

**Fig. 5:**
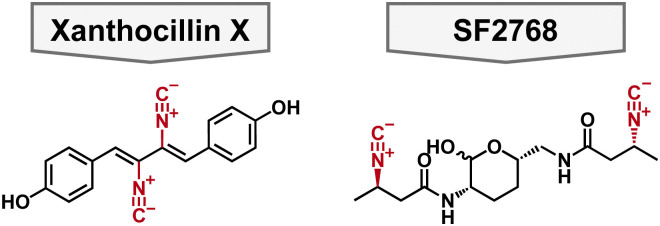
Structure of antibacterial chalkophore small molecules featuring a diisonitrile functional group colored in red.

In 1948, xanthocillin X was isolated from *Pencillium chrysogenum* and was reported to have broad spectrum antibacterial activity.^83,84^ To this end, it was briefly used as a topical antibacterial under the brand name Brevicid,^85,86^ however, due to incompatibility with serum and systemic toxicity its commercial use was terminated.^84,87^ Despite these flaws, xanthocillin X is a structurally interesting compound for chemists for its diisonitrile functionalities which are known to coordinate to a variety of metals (Fig. 5). Moreover, its mechanism of action was unknown making it a useful tool compound to study the potential of isonitriles as antibacterials. Specifically, xanthocillin X is effective against *A. baumannii* with an MIC of 0.25 μM, but its antibacterial activity is decreased against other clinically relevant pathogens, including *P. aeruginosa* with an MIC of 3 μM. ^88^ Xanthocillin X only significantly binds to Cu^2+^ of all the biologically relevant metals tested (Al, Co, Fe, Mn, Mg, Ni, and Zn). Furthermore, when produced by *Aspergillus fumigatus*, copper limitation increases the expression of the xanthocillin X suggesting that it is important for microbial metal homeostasis.^88^

In 2021, Hüber *et al.* aimed to determine xanthocillin X's mechanism of action. They generated spontaneous resistant mutants and performed affinity and activity-based proteomics in *A. baumannii*.^89^ These data showed various mutations in heme biosynthesis and changes in heme regulation; most interestingly, none of their data pointed to a protein target for xanthocillin X. To this end, it was hypothesized that the isonitrile moieties could bind the heme center thus leading to uncontrolled heme synthesis and subsequent cell death. This is the first of this type of mechanism of action reported and suggests that further development of xanthocillin X could lead to a novel antimicrobial compound.

### SF2768

SF2768 is a lipopeptide originally isolated from *Streptomyces* sp. SF2768 wherein it was reported to have antibacterial and antifungal activity against a wide range of pathogens.^90^ In 2017, it was isolated again from *Streptomyces thioluteus* and then heterologously expressed in *S. lividans.*^91^ It is characterized by the distinct diisonitrile moiety in addition to the central lactol ring (Fig. 5). In addition to the cyclized natural product, acyclic precursors were also isolated which give insights into the biosynthesis of the natural product. Given that SF2768 has the distinct isonitrile functional groups, it's metal binding abilities were examined, and it preferentially binds to Cu^+^ in a 2 : 1 ratio of ligand to metal. It was proposed that SF2768's mechanism of action would be disruption of metabolic processes reliant on a copper cofactor, such as cytochrome *C* oxidase.^91^ However, this was speculative based on metabolic processes that involve copper cofactors. Another study found that when bacteria, including *A. baumannii*, were treated with SF2768 their growth was inhibited in addition to an increase in reactive oxygen species (ROS).^92^ This could support the hypothesis that SF2768 targets a central pathway that increases the cells stress response which would explain the increase in ROS.

To aid in the study of SF2768, Tan and coworkers published the total synthesis of the natural product and the linear analogs, which allowed for the determination of the absolute stereochemistry of the natural product and highlighted the challenges of synthesizing the central lactol.^93^ Furthermore, Tan and coworkers supported previous work regarding the copper binding abilities of linear precursors of SF2768, and showed that SF2768 can bind to Zn^2+^, albeit more weakly than with Cu^+^. With the synthesis of SF2768 in hand, future researchers can probe the structure activity relationship and determine its potential as a Gram-negative antimicrobial.

## Antibiotic–metallophore conjugates and combinations

**Fig. 6:**
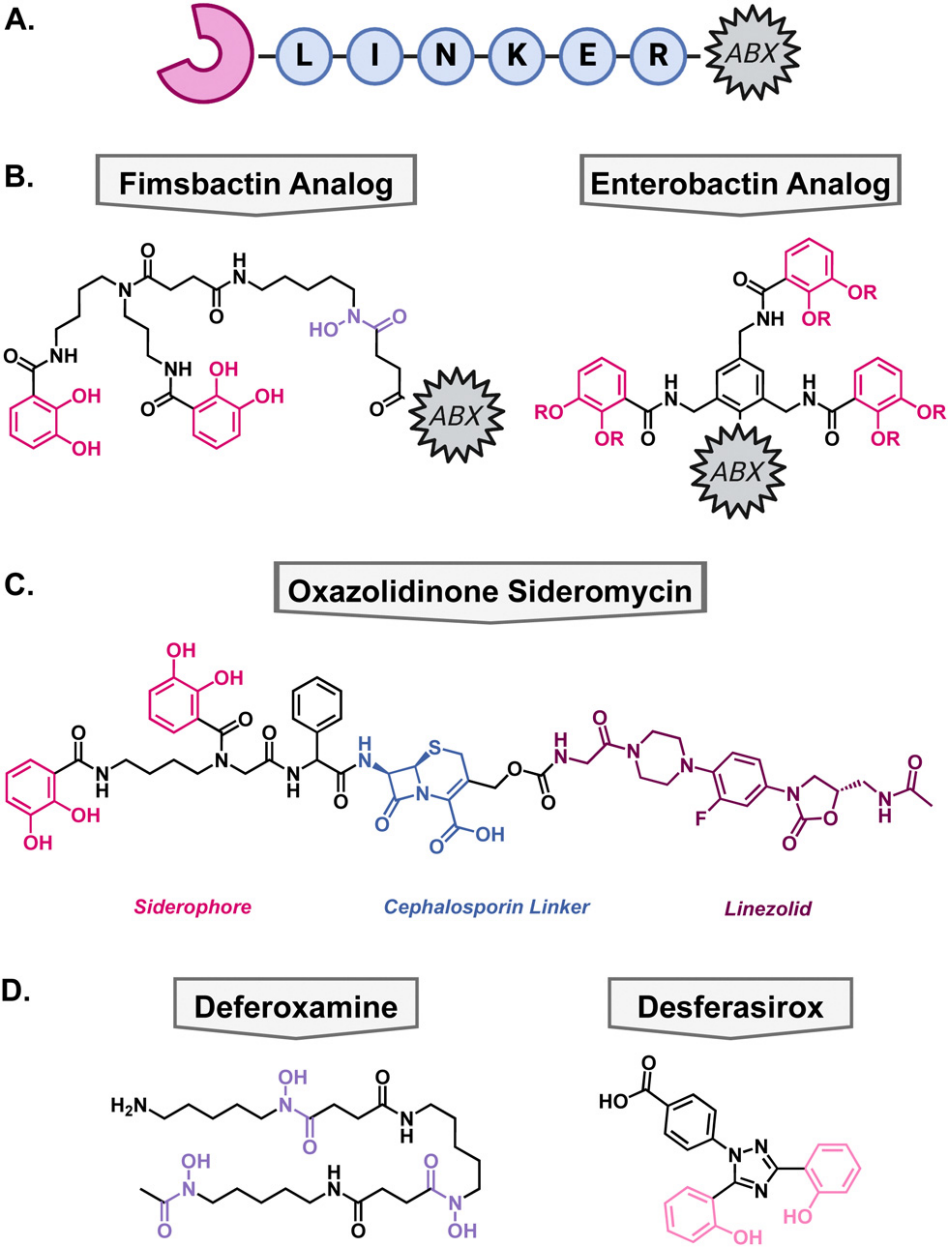
(A) Generic structure of a sideromycin wherein a metallophore (pink) is connected to an antibiotic (ABX, black) through a linker (blue). (B) Analogs of native siderophores fimsbactin and enterbactin for synthetic ease of conjugation to antibiotics. (C) Structure of siderophore–cephalosporin–oxazolidinone conjugate. (D) Structures of FDA approved iron chelators used in combination with tobramycin.

Another method to enhance the efficacy of current antibiotics is to increase their uptake into cells. To take advantage of some bacteria's promiscuous siderophore transporters, nature has combined metal binding moieties with antibacterial compounds. In the field, this is called a “Trojan-Horse” type mechanism since the antibiotic is being disguised by the metallophore's ability to bring the cell a necessary nutrient, usually iron. These types of compounds are called sideromycins. Generic sideromycins have three parts: a siderophore motif, a linker, and an antibacterial warhead (Fig. 6(A)). Each part requires intentional engineering and optimization, especially the linker as it cannot interact with the siderophore recognition site and may need to be cleaved intracellularly so that the antibiotic can reach its target. Medicinal chemists have employed a variety of approaches to synthesize novel sideromycins that are able to restore susceptibility to resistant bacteria or increase the scope for which the antibiotic can be used. Select examples of this strategy have been highlighted below.

### Natural sideromycins

Prior to synthetic chemists engineering synthetic sideromycins, nature was one step ahead. Of the sideromycins isolated, the most well-known are albomycin and salmycin. Albomycin was isolated from *Actinomyces subtropicus* and is the only isolated sideromycin that exhibits antibacterial activity towards some Gram-negative bacteria, such as *E. coli* and *Salmonella typhimurium*.^94,95^ It binds Fe^3+^*via* the hydroxamate moiety and a serine separates the nucleoside warhead whose intracellular target is aminoacyl-tRNA synthetases.^96^ Interestingly, this is not one of the classical antibiotic targets thus making albomycin an interesting tool compound to study the mechanism of aminoacyl-tRNA synthetase inhibition. Salmycin which was isolated from *Streptomyces violaceus* and employs an aminoglycoside warhead.^97^ However, it is not effective on Gram-negative bacteria therefore it is out of the scope of this review.

Of the less well known sideromycins, there are the microcins, ferrimycin, and danomycin. Microcin E492m is an enterobactin–peptide conjugate produced by *K. pneumoniae* RYC492 and shows potent antibacterial activity towards *E. coli*, likely due to the recognition of enterobactin by its native uptake machinery.^98^ However, what is more interesting about this sideromycin is its size: it is an 84mer peptide, highlighting the incredible size range for active sideromycins. Ferrimycin was isolated in the 1960's from *Streptomyces galilaeus*, however, there is limited knowledge on the scope of its antibacterial activity.^99,100^ In a high-throughput biosensor screen of natural products, ferrimycin was found to selectively inhibit protein biosynthesis in *Bacillus subtilis*.^101^ Shortly behind the isolation of ferrimycin, danomycin was isolated from the novel species *Streptomyces albaduncus* and has been compared to ferrimycin due to their similar antibacterial activities.^102,103^ Danomycin's mechanism of action and scope of activity, like many of these lesser known sideromycins, needs further investigation. Moreover, there unfortunately have not been any isolated sideromycins that are effective against *A. baumannii* or *P. aeruginosa* thus far which has led to a strong interest in the synthesis of novel metal binding antibiotics for these pathogens of interest.

### Siderophore derived antibiotic conjugates

Since Gram-negative bacteria are intrinsically resistant to many antibiotics, there has been a significant effort towards expanding the scope of Gram-positive antibiotics. Most Gram-positive antibiotics have an analogous molecular target in Gram-negative bacteria but are unable to access it due to the impermeability of the lipopolysaccharide and outer membrane as discussed previously.^104^ Therefore, conjugation to a Gram-negative siderophore could allow active uptake into the cell for the antibiotic to exert its mechanism of action. There are many accounts of native siderophores being conjugated to antibiotics which has been extensively review by Rayner *et al.*^105^

The Miller group has been at the forefront of synthetic sideromycin research and has designed a simplified analog of fimsbactin for facile conjugation to antibiotics of interest, usually through a simple amide coupling (Fig. 6(B)).^106^ As a model system, they used daptomycin as its large size and overall negative charge exclude it from passing the lipopolysaccharide and outer membrane.^106,107^ With this strategy, daptomycin conjugates have been shown to be effective against *A. baumannii* with MICs less than 1 μM.^107,108^ Additionally, beta-lactam antibiotics have also been a popular candidate for conjugation because of their inner membrane target so they can exert its antibacterial mechanism of action directly after active transport.^106^

More recently, the Brönstrup group designed a simplified analog of enterobactin to support the scalability and stability of these conjugates.^109^ To that end, they hypothesized that they could substitute the trilactone core of enterobactin with a benzene ring wherein the three catechol arms can bind iron, and a fourth substitution allows for antibiotic conjugation (Fig. 6(B)). The ampicillin conjugate achieved a 90 nM MIC against *A. baumannii* and a daptomycin conjugate had an MIC of 4.4 μM highlighting the efficacy of this strategy. These simplified antibiotic conjugates have yet to show antibacterial activity towards *P. aeruginosa*, despite intracellular accumulation. Current work is focused on exploiting siderophore transport machinery as a novel antibacterial target.^110^

### Siderophore–cephalosporin–oxazolidinone conjugate

One of the downfalls to synthetic antibiotic–metallophore conjugates is that the antibiotic's activity can be hindered by the covalent modification to the metallophore. This can prevent the antibiotic from reaching its molecular target; therefore, many of these modifications have focused on using antibiotics with inner membrane mechanisms of action, such as daptomycin and beta-lactams. Some strategies to overcome this have employed cleavable moieties to free the antibiotic after transport into the periplasm. These were inspired by the intracellular cleavage of albomycin and salmycin by a peptidase or intramolecular esterification, respectively. However, synthetic sideromycins with cleavable moieties are prone to degradation before reaching the bacteria.

When beta-lactam antibiotics are used in sideromycins, it has been noted that they can still be susceptible to inactivation by beta-lactamases. Given the abundance of beta-lactamases in Gram-negative pathogens, it was hypothesized that this could be used as a cleavable linker to deliver antibiotics with a cytosolic target that would normally suffer from poor permeability and/or swift efflux. To test this hypothesis, Miller and coworkers designed a novel oxazolidinone conjugate wherein the catechol would facilitate uptake into the cell, and the cephalosporin would be cleaved by native beta lactamases thus freeing the oxazolidinone to enter the cytosol (Fig. 6(C)).^111^ After synthesis of the oxazolidinone conjugate, they tested their hypothesis by tracking the compound in the presence of a cephalosporinase *via* LC-MS. They found that the cephalosporin was readily cleaved in the presence of beta-lactamases *in vitro* and then moved into antibacterial testing to determine if this would translate *in vivo*. The conjugate was tested against *A. baumannii*, *P. aeruginosa*, and *E. coli* and exhibited MICs of 0.2–6.25 μM against the pathogens. Furthermore, they supplemented one of the *A. baumannii* strains with a plasmid to encode for the addition of cephalosporinase and the compounds remained active. This is important as clinical isolates of these Gram-negative pathogens have high levels of these enzymes and highlights the utility of this strategy for future antibiotic development.

### Tobramycin in combination with FDA-approved iron chelators

In addition to attaching metal binding moieties to molecules, metallophores have also been used in combination with antibiotics. One such case is with tobramycin, which is the standard of care for patients with chronic *P. aeruginosa* infections as a consequence of the genetic disorder cystic fibrosis (CF).^112^ One reason that these patients have recurrent infections is the development of biofilms wherein bacteria become increasingly tolerant to antibiotics. As mentioned earlier, iron is important for the regulation of virulence factors such as biofilms, therefore, disrupting iron homeostasis in combination with antibiotic treatment could be beneficial. To determine if tobramycin could be made more effective when iron is sequestered away from *P. aeruginosa*, Moreau-Maquis and colleagues used it in combination with the FDA-approved iron chelators deferoxamine mesylate (DFO) and desferasirox (DSX).^113^ These drugs are normally used for the treatment of chronic iron overload or acute iron intoxication but have not been considered as antibiotic adjuvants.^114,115^ When biofilms were grown on plastic surfaces, the combination treatment of tobramycin and either iron chelators did not disturb the biofilm more than tobramycin alone. However, this was not the case when they changed the system to CF airway epithelial cells. In this system, DFO, DSX, and tobramycin inhibited biofilm formation by 47%, 99%, and 97%, respectively. When in combination with tobramycin, DFO did not show a difference, but DSX enhanced the activity of tobramycin for inhibition of biofilm formation. Unfortunately, the combination of the iron chelators and tobramycin was not able to disperse mature biofilms to a greater extent than tobramycin alone which limits the clinical relevance of the study. Furthermore, the study focused on the quantification of biomass without determining the viability of the cells which is the true reason for recurrent infections. These data support the hypothesis that iron is an important nutrient for the establishment of biofilms, but disruption of iron homeostasis is not the most effective strategy for the dispersal of mature biofilms.

## Metal binding as a clinical strategy

Taking a page from nature's book, medicinal chemists have tried to bring metallophore antibiotics into the clinic since they show great success in *in vitro* and *in vivo* studies. However, this has been a challenge not only because of the regulatory constraints of antibiotic development but cross-toxicity when brought into clinical trials. Up to this point, all the metallophore antibiotics that have gone to clinical trials have been derivatives of beta-lactams due to the easier access of the molecular target in the periplasm. Although there have been many failed attempts to bring metallophore antibiotics into the clinic, there is one success story that gives a strong example for future clinical development.

### Three failed attempts to bring metallophores into the clinic

To date, only four metallophore antibiotics have been brought into clinical trials – of which three did not make it past phase I. This not only highlights the difficulty of bringing a molecule from “proof-of-principle” to a preclinical candidate but also the high rate of failure in antibiotic development.

**Fig. 7:**
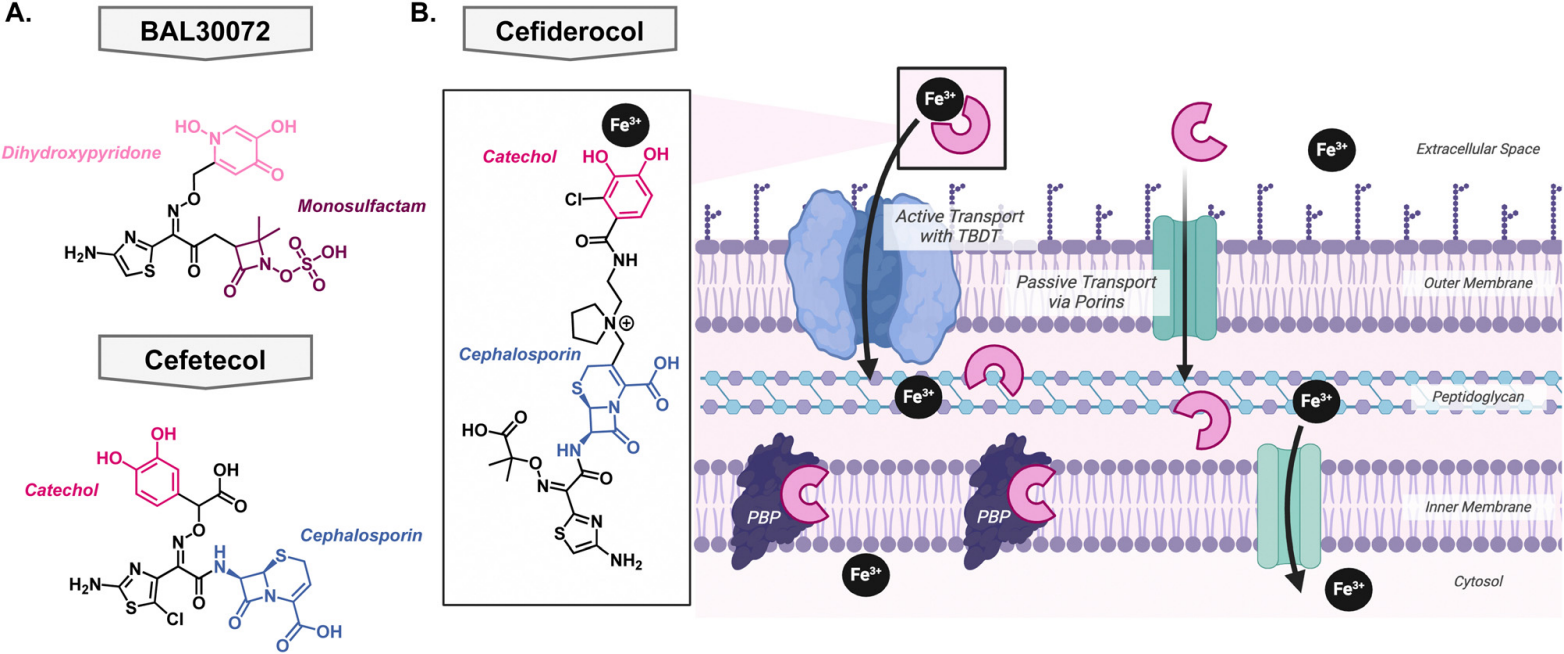
(A) Structures of iron binding antibiotics BAL30072 and cefetecol which did not pass clinical trials. (B) Structure of cefiderocol and its mechanism of action of as both an iron complex and canonical cephalosporin.

The first of these attempts was pursued by Basilea Pharmaceutica who developed BAL30072, a monosulfactam conjugated to a hydroxypyridone with the goal to act as a catechol isostere Fig. 7(A).^116,117^ It shows broad Gram-negative activity towards *Acinetobacter* species, *P. aeruginosa*, and *Enterobacteriaceae* and displayed low development of resistance.^118^ The most common mechanism of resistance was over expression on beta-lactamases rather than in the TonB protein or siderophore recognition sites.^119^ Despite the promising *in vitro* activity, Basilea Pharmaceutica suspended the development in phase I clinical trials. There has also been development of siderophore antibiotics with catechol functional groups, however, these early *in vitro* investigations did not translate to potent *in vivo* activity. Unfortunately, the catechol was a metabolic hotspot and would be methylated by catechol *O*-methyltransferase rendering the compound unable to effectively bind iron for uptake into the pathogen of interest. This was the case for the drug candidate cefetecol and GSK3342830; their development was terminated during phase I clinical trials Fig. 7(A).^116^ The structure of GSK3342830 has not been disclosed. Further medicinal chemistry efforts revealed that by tuning the p*K*_a_ of the phenol group, they can prevent methylation and increase its metabolic stability. Therefore, current catechol-containing metallophores have an electron-withdrawing halogen at the adjacent position to decrease the p*K*_a_ of the phenol and maintain its ability to bind iron.

### Success story: cefiderocol

After learning from the failed attempts to bring a metallophore antibiotic through clinical trials, Shionogi & Co. finally succeeded. In 2019, cefiderocol received FDA-approval as the first metallophore antibiotic Fig. 7(B). Originally its approval was for the treatment of complicated urinary tract infections, but it was expanded in 2020 to hospital-acquired bacterial pneumonia and ventilator-associated bacterial pneumonia from Gram-negative bacteria,^120^ likely due to the COVID-19 pandemic. It contains the same antibacterial warhead as ceftazidime, which is stable to many beta lactamases, and the metal binding motif is an electron withdrawn catechol Fig. 7(B). As described above, this is an important feature as it ensures that the catechol is not a metabolic liability and can bind iron effectively. Importantly, its linker is pyrrolidinium which helps improve antibacterial activity and stability to beta-lactamases. A thorough account of the structure activity relationship of cefiderocol was reported by Aoki and colleagues.^121^ Cefiderocol can access its target *via* passive transport, like other cephalosporins, but it has the advantage of being able to bind iron with the catechol moiety which allows active uptake into the cell *via* the TonB dependent transports that are used for siderophores Fig. 7(B).^122^ At this point, it can release the iron into the periplasm and bind the penicillin binding proteins to exert its bacterial killing mechanism.^123^

Since the approval of cefiderocol by the FDA, its use has been expanded from solely prescribed for complicated urinary tract infections to also include hospital-acquired bacterial pneumonia and ventilator-associated bacterial pneumonia. As its use increased, bacteria developed resistance to the antibiotic through the upregulation of efflux pumps and mutations in siderophore transport systems; these mechanisms have been previously reviewed by Karakonstantis *et al.*^124^ This raises the question of whether cefiderocol could be used alongside other antibacterials to slow the emergence of resistance. One study looked at the combination with colloidal bismuth citrate which is used in the treatment of *Helicobacter pylori.*^125^ Bismuth can compete with iron for cellular uptake so it was hypothesized that it could be used in combination with cefiderocol to provide a dual killing mechanism thus preventing rapid resistance development.^126^ The combination showed a synergistic relationship in both *in vitro* and *in vivo* models. Clinically, cefiderocol has been used in combination with polymyxins and beta-lactam/beta-lactamase inhibitor therapies, however, general trends have not been revealed.^127–131^ This can be attributed to the complex resistance profiles and drug interactions thus necessitating further research.

## Conclusion and outlook

As bacteria become increasingly resistant to current antibiotic treatments, it is crucial that there is constant innovation in antimicrobial development. Some of the current efforts discussed herein include increasing antibiotic uptake in Gram-negative bacteria and the development of virulence inhibitors to “disarm” pathogens. In recent years, chemical modification to antibiotics has been successful in increasing the uptake of antibiotics in concerning Gram-negative pathogens such as *A. baumannii* and *P. aeruginosa*, however, conclusions regarding the structure activity relationship of these compounds lack precision. Additionally, increased compound accumulation has been observed when antibiotics are conjugated to siderophores, and this is a proven strategy given the use of cefiderocol in the clinic.

To target pathogen virulence, disruption of metal homeostasis has been one strategy as it supports several virulence factors and overall cell survival. For decades, iron has been the main metal of interest, however, recently key roles for other metals such as copper, zinc, and cobalt have been discovered which can lead to new ways of thinking about disrupting metal homeostasis. To leverage other metals in antibacterials, it will be important to fully understand their mechanisms of acquisition, transport, and regulation.

In addition to the native metallophores that have been discovered, advances in genome mining have led to an increase in understanding biosynthetic gene clusters and the metabolites that they produce. Some of these metabolites have been found to bind metals but how that relates to their function for the producing organism is vague. These metabolites could also function as antibacterials, but many have not been tested against relevant pathogens. Furthermore, the isolation of malleonitrone highlights the importance of looking at interspecies chemical warfare for novel antibacterials.

Novel structures of antibacterial metallophores suggest alternative mechanisms of action with unique targets, like the discovery of the mechanism of action of xanthocillin X or the unique selectivity of maipomycin A. Beyond the molecules discussed herein, there's a wide library of metallophores that show activity against Gram-positive pathogens but lack Gram-negative activity; therefore, it would be useful to marry the approaches of synthetic sideromycins and the eNTRY rules to target these challenging pathogens. This type of approach would expand the structural activity relationship knowledge for compounds that are effective against Gram-negative bacteria.

Furthermore, synthetic sideromycins are opening doors to treating bacteria with antibiotics that they have not been exposed to before which could restore susceptibility for some of these highly resistant strains. Moreover, their resistance mechanisms can be exploited to introduce new classes of antibiotics to intrinsically resistant pathogens, such as the case with the oxazolidinone–cephalosporin sideromycin. Moreover, there is a proof-of-principle that metal binding can be an effective antibacterial strategy as seen by cefiderocol. The future of the field will continue to discover novel metal binding motifs while searching for natural products, innovate new ways for sideromycin cleavage, and continue to push the boundaries on how we can out-smart bacteria them?

## Author contributions

M. M. Golden: conceptualization, writing – original draft, review & editing. A. C. Heppe: writing – original draft, review & editing. C. L. Zaremba: writing – review & editing. W. M. Wuest: conceptualization, funding acquisition, supervision, writing – review & editing. All authors have approved of the final draft of the manuscript.

## Conflicts of interest

There are no conflicts to declare.

## Data Availability

No primary research results, software or code have been included and no new data were generated or analysed as part of this review.
